# London Prices of Drugs.—Feb. 26

**Published:** 1814-04

**Authors:** 


### LONDON PRICES OF DRUGS.—Feb. 26.

*(To be continued regularly.)*

| | £ | s. | d. | £ | s. | d. | per |
|---|---|---|---|---|---|---|---|
| Aloes Barbadoes, from | 20 | 10 | 0 to | 21 | 0 | 0 | C. |
| ——— Cape | 10 | 0 | 0.. | 11 | 0 | 0 | — |
| ——— Succotrina | 20 | 0 | 0.. | 0 | 0 | 0 | — |
| ——— Epatica or E.I. | 10 | 0 | 0.. | 12 | 0 | 0 | — |
| Angelica Root | 12 | 0 | 0.. | 13 | 0 | 0 | — |
| Alkanet Root | 8 | 0 | 0.. | 0 | 0 | 0 | — |
| Antimony Crude | 3 | 15 | 0.. | 4 | 0 | 0 | — |
| Aqua Fortis | S. | 0 | 8..D. | 1 | 1 | | lb |
| Arrow Root fine | 0 | 2 | 0.. | 0 | 3 | 6 | — |
| ——— ordinary | 0 | 1 | 0.. | 0 | 1 | 6 | — |
| Arsenic Red | 5 | 0 | 0.. | 5 | 5 | 0 | C. |
| ——— White | 3 | 10 | 0.. | 3 | 12 | 0 | — |
| Balsam Capivi | 0 | 5 | 6.. | 0 | 0 | 0 | lb. |
| ——— Peru | 0 | 19 | 0.. | 1 | 0 | 0 | — |
| ——— Tolu | 0 | 12 | 0.. | 0 | 13 | 0 | — |
| Bark Jesuit Com. | 0 | 3 | 6.. | 0 | 4 | 0 | — |
| ——— Second | 0 | 6 | 0.. | 0 | 7 | 0 | — |
| ——— Quil or best | 0 | 8 | 0.. | 0 | 10 | 0 | — |
| ——— Red | 0 | 7 | 0.. | 0 | 10 | 0 | — |
| ——— Yellow | 0 | 4 | 6.. | 0 | 5 | 0 | — |
| Borax refined E.I. | none. | | | | | | |
| ——— English | 0 | 3 | 6.. | 0 | 3 | 8 | lb. |
| ——— unrefined or Tinc. | 10 | 0 | 0.. | 11 | 0 | 0 | C. |
| Camphire refined | 0 | 8 | 9.. | 0 | 9 | 0 | lb. |
| ——— unrefined | 28 | 0 | 0.. | 32 | 0 | 0 | C. |
| Cantharides | 0 | 11 | 0.. | 0 | 11 | 3 | — |
| Cardamoms (best) | 0 | 7 | 6.. | 0 | 0 | 0 | lb. |
| Cassia Buds | 12 | 0 | 0.. | } | 0 | 0 | C. |
| ——— Fistula W.I. | 12 | 0 | 0.. | } in bond | | | — |
| ——— Lignea | 12 | 0 | 0.. | } | 0 | 0 | — |
| Castorum American | 2 | 10 | 0.. | 2 | 15 | 0 | lb. |
| ——— Russia | 11 | 0 | 0.. | 0 | 0 | 0 | — |
| Castor Oil per bottle 1½ lb. | 0 | 3 | 9.. | 0 | 4 | 3 | bo |
| Cocculus Indicus | 3 | 0 | 0.. | 0 | 0 | 0 | C. |
| Colocynth Turkey | 0 | 5 | 0.. | 0 | 5 | 6 | lb. |
| Columbo Root | 2 | 15 | 0.. | 3 | 0 | 0 | C. |
| Cream of Tartar | 1 | 10 | 0.. | 9 | 0 | 0 | — |
| Gallangal East-India | 5 | 0 | 0.. | 0 | 0 | 0 | C. |
| Gentian Root | 3 | 15 | 0.. | 4 | 0 | 0 | — |
| Ginseng | 0 | 1 | 6.. | 0 | 0 | 0 | lb. |
| Grains of Guinea | 5 | 10 | 0.. | 6 | 0 | 0 | C. |
| Gum-Ammo. Drop | 30 | 0 | 0.. | 0 | 0 | 0 | — |
| ——— Lump | 8 | 0 | 0.. | 9 | 0 | 0 | — |
| ——— Animi | 3 | 10 | 0.. | 7 | 10 | 0 | — |
| ——— Arabic E.I. | 2 | 0 | 0.. | 4 | 0 | 0 | — |
| ——— Turkey Fine | 8 | 10 | 0.. | 9 | 9 | 0 | — |
| ——— Barbary | 7 | 7 | 0.. | 8 | 0 | 0 | — |
| ——— Assafœtida | 10 | 0 | 0.. | 15 | 0 | 0 | — |
| ——— Benjamin | 8 | 10 | 0.. | 35 | 0 | 0 | — |
| ——— Cambogium | 20 | 0 | 0.. | 26 | 0 | 0 | — |
| ——— Copal scraped | 0 | 3 | 0.. | 0 | 4 | 6 | lb. |
| ——— Galbanum | 16 | 0 | 0.. | 20 | 0 | 0 | C. |

| | £ | s. | d. | £ | s. | d. | per |
|---|---|---|---|---|---|---|---|
| Gum Guaiacum, from | 0 | 2 | 6 to | 0 | 3 | 0 | lb. |
| ——— Mastic | 0 | 4 | 6.. | 0 | 4 | 9 | — |
| ——— Myrrh | 15 | 0 | 0.. | 18 | 0 | 0 | C. |
| ——— Olibanum | 6 | 0 | 0.. | 7 | 0 | 0 | — |
| ——— Oppoponax | 80 | 0 | 0.. | 0 | 0 | 0 | — |
| ——— Sandrac | 9 | 0 | 0.. | 10 | 0 | 0 | — |
| ——— Seneca Garbled | 5 | 2 | 0.. | 5 | 5 | 0 | — |
| ——— Tragacanth | 30 | 0 | 0.. | 35 | 0 | 0 | — |
| Jalap | 0 | 5 | 0.. | 0 | 5 | 3 | lb. |
| Ipecacuanha | 1 | 2 | 0.. | 1 | 3 | 0 | — |
| Isinglass Book | 0 | 10 | 6.. | 0 | 11 | 0 | — |
| ——— Leaf | 0 | 10 | 6.. | 0 | 11 | 0 | — |
| ——— Long Staple | 0 | 10 | 6.. | 0 | 11 | 0 | — |
| ——— Short Staple | 0 | 10 | 6.. | 0 | 11 | 0 | — |
| Manna Flakey | 0 | 5 | 6.. | 0 | 6 | 0 | — |
| ——— Sicily in Sorts | 0 | 3 | 3.. | 0 | 4 | 0 | — |
| Musk China | 0 | 18 | 0.. | 1 | 0 | 0 | oz. |
| Nux Vomica | 3 | 0 | 0.. | 3 | 10 | 0 | C. |
| Oil of Vitriol | 0 | 0 | 3¾ | 0 | 0 | 0 | lb. |
| Opium East-India | 0 | 19 | 6.. | 1 | 0 | 0 | — |
| ——— Turkey | 1 | 9 | 0.. | 1 | 10 | 0 | — |
| Pink Root | 0 | 4 | 0.. | 0 | 4 | 6 | — |
| Quicksilver | 0 | 4 | 10.. | 0 | 0 | 0 | — |
| Rhubarb East-India | 0 | 3 | 0.. | 0 | 5 | 0 | — |
| ——— Russia | 0 | 18 | 0.. | 1 | 0 | 0 | — |
| Saffron Spanish | 2 | 5 | 0.. | 2 | 10 | 0 | — |
| ——— French | 1 | 15 | 0.. | 2 | 0 | 0 | — |
| Sago | 7 | 0 | 0.. | 8 | 0 | 0 | C. |
| Sal. Ammoniac | 10 | 10 | 0.. | 11 | 0 | 0 | — |
| Sarsaparilla | 0 | 3 | 0.. | 0 | 3 | 6 | lb |
| Sassafras | 8 | 0 | 0.. | 9 | 0 | 0 | T. |
| Scammony Aleppo | 1 | 6 | 0.. | 1 | 7 | 0 | lb. |
| ——— Smyrna | 0 | 10 | 0.. | 0 | 12 | 0 | — |
| Senna | 0 | 3 | 0.. | 0 | 0 | 0 | — |
| Seeds Anni Alicant | 6 | 0 | 0.. | 6 | 10 | 0 | C. |
| ——— Coriander English | 1 | 10 | 0.. | 1 | 16 | 0 | — |
| ——— Cummin | 7 | 0 | 0.. | 7 | 10 | 0 | — |
| ——— Fenugreek | 1 | 10 | 0.. | 1 | 13 | 0 | — |
| Shellack | 7 | 0 | 0.. | 8 | 8 | 0 | — |
| Sticklack | 6 | 0 | 0.. | 8 | 0 | 0 | — |
| Snake Root | 0 | 0 | 0.. | 0 | 0 | 0 | lb. |
| Soap Castile or Spanish | 12 | 0 | 0.. | 14 | 0 | 0 | C. |
| Spermaceti refined | 0 | 2 | 7.. | 0 | 0 | 0 | lb. |
| Tamarinds West-India | 8 | 10 | 0.. | 9 | 0 | 0 | C. |
| Tapioca Lisbon | 0 | 0 | 8.. | 0 | 1 | 0 | lb. |
| Turmeric Bengal | 5 | 10 | 0.. | [illegible] | 0 | 0 | C. |
| ——— China | 6 | 10 | 0.. | 7 | 0 | 0 | — |
| ——— West-India | 8 | 0 | 0.. | 9 | 0 | 0 | — |
| Verdigris Wet | 0 | 3 | 6.. | 0 | 0 | 0 | lb. |
| ——— Dry | 0 | 5 | 6.. | 0 | 5 | 8 | — |
| ——— Crystallized | 0 | 8 | 6.. | 0 | 8 | 9 | — |
| Vitriol Roman | 0 | 0 | 7.. | 0 | 0 | 0 | — |
| ——— Foreign white | 4 | 0 | 0.. | 4 | 10 | 0 | C. |

### OBSERVATIONS.

At recent Sales of Merchandize by Auction, Messrs. Bowden and Tucker sold, on the 21th of March, 419 chests Pale and Peruvian Bark, bonded, 1s. 6d. to 5s. 10d. per lb.—20 chests Yellow Ditto, bonded, 4s. 1d. to 4s. 3d. per lb.—9 chests Red Ditto, bonded, 5s. 6d. to 9s. 3d. per lb.—7 chests Yellow Ditto, duty paid, 5s. 1d. to 5s. 10d. per lb.—24 chests Rhubarb, 1s. 2d. to 9s. 1d per lb.—15 casks Balsam Capivi, duty paid, 6s. 3d. to 6s. 6d. per lb.—2 bales Pellitory Root, 20s. per lb.

